# Exploring an algorithm to harmonize International Obesity Task Force and World Health Organization child overweight and obesity prevalence rates

**DOI:** 10.1111/ijpo.12905

**Published:** 2022-02-22

**Authors:** Tim J. Cole, Tim Lobstein

**Affiliations:** ^1^ Population, Policy and Practice Research and Teaching Programme University College London Great Ormond Street Institute of Child Health London UK; ^2^ World Obesity Federation London UK; ^3^ Centre for Health Economics & Policy Innovation Imperial College London UK

**Keywords:** harmonization, IOTF, obesity, overweight, prevalence, WHO

## Abstract

**Background:**

The International Obesity Task Force (IOTF) and World Health Organization (WHO) body mass index (BMI) cut‐offs are widely used to assess child overweight, obesity and thinness prevalence, but the two references applied to the same children lead to different prevalence rates.

**Objectives:**

To develop an algorithm to harmonize prevalence rates based on the IOTF and WHO cut‐offs, to make them comparable.

**Methods:**

The cut‐offs are defined as age‐sex‐specific BMI *z*‐scores, for example, WHO +1 SD for overweight. To convert an age‐sex‐specific prevalence rate based on reference cut‐off A to the corresponding prevalence based on reference cut‐off B, first back‐transform the *z*‐score cut‐offs $z_{A}$ and $z_{B}$ to age‐sex‐specific BMI cut‐offs, then transform the BMIs to *z*‐scores $z_{B,A}$ and $z_{A,B}$ using the opposite reference. These *z*‐scores together define the distance between the two cut‐offs as the *z*‐score difference ${\mathrm{dz}}_{A,B}=\frac{1}{2}\left(z_{B}-z_{A}+z_{A,B}-z_{B,A}\right)$. Prevalence in the target group based on cut‐off A is then transformed to a *z*‐score, adjusted up or down according to ${\mathrm{dz}}_{A,B}$ and back‐transformed, and this predicts prevalence based on cut‐off B. The algorithm's performance was tested on 74 groups of children from 14 European countries.

**Results:**

The algorithm performed well. The standard deviation (SD) of the difference between pairs of prevalence rates was 6.6% (*n* = 604), while the residual SD, the difference between observed and predicted prevalence, was 2.3%, meaning that the algorithm explained 88% of the baseline variance.

**Conclusions:**

The algorithm goes some way to addressing the problem of harmonizing overweight and obesity prevalence rates for children aged 2–18.

## 1 INTRODUCTION

Child obesity has long been a worldwide public health concern.^1^ Child body mass index (BMI) has now plateaued in many high‐income countries, but it continues to rise elsewhere.^2^ The universally accepted definitions of overweight and obesity in adults are BMI 25+ and 30+ kg/m^2^, respectively,^3^ and the changing prevalence of overweight and obesity across countries and regions over time has been widely documented, a recent study summarizing rates in 200 countries.^4^ BMI is also used to assess thinness, defined as BMI less than 18.5 kg/m^2^.

The assessment of overweight, obesity and thinness in children is more complex than in adults. As a measure, BMI is equally valid, but because children grow in height and weight, child BMI needs adjusting for age and sex using a suitable reference.^5^ There are two widely used international child BMI references, the International Obesity Task Force (IOTF) cut‐offs,^6^, ^7^, ^8^ and the World Health Organization (WHO) growth standard and reference.^9^, ^10^ The US Centers for Disease Control and Prevention (CDC) reference is also used,^11^ and all three are endorsed by the European Child Obesity Group (ECOG).^12^ The references convert BMI to an age–sex‐adjusted *z*‐score, and the categories of overweight, obesity and thinness are defined as the child's *z*‐score lying above (or for thinness, below) the relevant *z*‐score cut‐off (see Table 1).

**TABLE 1:** BMI *z*‐score cut‐off definitions of child thinness, overweight and obesity according to the IOTF, WHO and CDC references^12^

Reference	Sex	Thinness^a^	Overweight^b^	Obesity
IOTF 2–18 years	Boys	−1.01, −1.88, −2.56^c^ (18.5, 17, 16)^d^	+1.31 (25)^d^	+2.29 (30)^d^
	Girls	−0.98, −1.79, −2.44^c^ (18.5, 17, 16)^d^	+1.24 (25)^d^	+2.19 (30)^d^
WHO 0–5 years^e^	Both	−2	+2	+3
WHO 5–19 years	Both	−2	+1	+2
CDC 2–20 years	Both	−1.64 (5th centile)	+1.04 (85th centile)	+1.64 (95th centile)

Abbreviations: BMI, body mass index; CDC, Centers for Disease Control; IOTF, International Obesity Task Force; WHO, World Health Organization.

^a^ Some definitions use the terms ‘wasting’ or ‘underweight’ rather than ‘thinness’.

^b^ Some studies include obesity prevalence in overweight prevalence whereas others exclude it.

^c^ Cut‐offs for IOTF thinness grades 1, 2 and 3, respectively.

^d^ Value of BMI at age 18 on which the IOTF *z*‐score is based.

^e^ WHO 0–5 years uses weight‐for‐height *z*‐score rather than BMI *z*‐score; the two are highly correlated^21^, ^29^ but distinct.^30^

The need for a BMI reference complicates the comparison of child prevalence rates across studies; different references applied to the same children lead to different prevalence rates.^13^, ^14^, ^15^, ^16^ This means that meta‐analyses of child prevalence studies cannot combine results based on different references—they need reporting separately. For example, two very large studies from the NCD Risk Factor Collaboration and the Global Burden of Disease restricted themselves to prevalence rates based on just one reference, respectively WHO and IOTF.^2^, ^17^ This represents an alarming waste of research effort. To work around it the journal *Pediatric Obesity* requests authors to report prevalence rates ‘*using both the IOTF and WHO definitions*’.^18^

This is an issue of *data harmonization*, the need to make prevalence rates comparable across studies. The same issue arose when WHO wanted to compare prevalence rates for nutritional indicators based on the 1977 national center for health statistics (NCHS) reference^19^ and the 2006 WHO growth standard.^20^ They developed a logistic regression algorithm to do the conversion.^21^ Paediatric Obesity has such an algorithm in mind when it tells authors, ‘*sufficient numbers of published studies which report [IOTF and WHO] prevalence values will be needed to generate the algorithms to estimate one from the other*’.^18^

In general, such algorithms have two weaknesses. First, they depend on the data used to estimate the regression equations, so they cannot reliably be applied to other data—they are not *generalizable*; and second, being regression‐based they are not *reversible*: they convert from reference A to reference B but not from B to A. The WHO algorithm was designed to convert from NCHS to WHO, but prevalence rates based on IOTF and WHO need to be interchangeable, that is, converting from one to the other and then back again should return the original value. What is needed is an algorithm to convert between prevalence rates that are both generalisable and reversible.

The aim of the study is to explore such a method for harmonizing prevalence rates for nutritional indicators across growth references, with a particular focus on overweight, obesity and thinness with IOTF, WHO and CDC.

## 2 METHODS

### 2.1 LMS method

The three BMI references, IOTF, WHO and CDC, are all based on the LMS method. This transforms BMI to a *z*‐score adjusting for skewness in the BMI distribution using three age‐sex‐specific parameters—L the Box‐Cox power, M the median and S the coefficient of variation.^22^, ^23^

### 2.2 The algorithm

Consider two references A and B (selected from IOTF, WHO and CDC), with *z*‐score cut‐offs $z_{A}$ and $z_{B}$, respectively (see Table 1). Each reference and *z*‐score cut‐off together define a corresponding age‐sex‐specific BMI cut‐off. The BMI cut‐off for reference A can also (for given age and sex) be expressed as a *z*‐score based on reference B; this *z*‐score is called $z_{A,B}$, while that for the BMI cut‐off based on B and *z*‐score based on A is $z_{B,A}$. The *z*‐scores $z_{A}$ and $z_{B,A}$ both represent the BMI cut‐off for reference A, and their average $\bar{z_{A}}$ is a symmetric estimate of the *z*‐score cut‐off for A. The same holds for $\bar{z_{B}}$ the average of $z_{B}$ and $z_{A,B}$ for reference B. So, expressed on a common *z*‐score scale, the difference ${\mathrm{dz}}_{A,B}$ between the A and B cut‐offs is given by:

(1) $${\mathrm{dz}}_{A,B}=\bar{z_{B}}-\bar{z_{A}}=\frac{1}{2}\left(z_{B}+z_{A,B}\right)-\frac{1}{2}\left(z_{A}+z_{B,A}\right)=\frac{1}{2}\left(z_{B}-z_{A}+z_{A,B}-z_{B,A}\right).$$

Overweight and obesity correspond to BMI in the upper tail of the distribution, where $z_{A}$ is positive, while for thinness in the lower tail $z_{A}$ is negative. Based on reference A, the prevalence p of BMI beyond $z_{A}$ is given by $p=\Phi \left(-\mathrm{sign}\left(z_{A}\right)\times z\right)$, where Φ is the cumulative normal distribution; this expression gives the appropriate tail area whether for thinness or overweight–obesity.

Equally one can convert prevalence to a *z*‐score. *Z*‐score Z corresponding to prevalence p is given by $Z=-\mathrm{sign}\left(z_{A}\right)\Phi^{-1}\left(p\right)$, where $\Phi^{-1}$ is the inverse cumulative normal distribution. Capital Z here emphasizes that the *z*‐score is not a reference cut‐off but is calculated from a prevalence rate.

Consider now a target group of children, of given sex and mean age, whose BMI distribution is known only in terms of its prevalence rates $p_{A}$ and $p_{B}$ beyond the BMI cut‐offs for references A and B, respectively. The aim of the algorithm is to predict one of these rates from the other. (To validate the algorithm both rates need to be known, but in general use one of them will be unknown.) Each prevalence can be converted to a *z*‐score: $Z_{A}=-\mathrm{sign}\left(z_{A}\right)\Phi^{-1}\left(p_{A}\right)$ and $Z_{B}=-\mathrm{sign}\left(z_{B}\right)\Phi^{-1}\left(p_{B}\right)$. Their difference $dZ_{A,B}=Z_{B}-Z_{A}$ is an estimate of the distance between the A and B cut‐offs.

Now comes the key assumption. Assume that all three BMI distributions (reference distributions A and B and the [unknown] distribution for the target group of children) are broadly the same in terms of L, M and S, and that they differ only in terms of a shift on the *z*‐score scale. On this basis, the two estimates of the *z*‐score difference can be set equal:

(2) $$dZ_{A,B}={\mathrm{dz}}_{A,B}.$$

The aim of the paper is to test this assumption.

To predict $p_{B}$ from $p_{A}$ using (2), first convert $p_{A}$ to $Z_{A}$, then shift it by ${\mathrm{dz}}_{A,B}$ and convert it back to $p_{B}$, as follows:

(3a) $$p_{B}=\Phi \left(-\mathrm{sign}\left(z_{B}\right)\times \left(Z_{A}+{\mathrm{dz}}_{A,B}\right)\right)+\varepsilon_{B}$$

where $\varepsilon_{B}$ is the error of prediction. To predict $p_{A}$ from $p_{B}$ apply the same formula in reverse, noting that ${\mathrm{dz}}_{B,A}=-{\mathrm{dz}}_{A,B}$:

(3b) $$p_{A}=\Phi \left(-\mathrm{sign}\left(z_{A}\right)\times \left(Z_{B}+{\mathrm{dz}}_{B,A}\right)\right)+\varepsilon_{A}$$

Equation (2) constrains the algorithm to be reversible, depending only on the sign of ${\mathrm{dz}}_{A,B}$. This means that the prediction error $\varepsilon_{A}=p_{A}-E\left(p_{A}\right)$ is equal and opposite in sign to $\varepsilon_{B}=p_{B}-E\left(p_{B}\right)$ as measured on the *z*‐score scale (where $E\left(.\right)$ indicates the expected value), but slightly different on the prevalence scale due to the nonlinear *z*‐score‐prevalence conversion. The performance of the algorithm is best judged by comparing the standard deviation (SD) of $\varepsilon_{A}$ (and $\varepsilon_{B}$) to the SD of $p_{A}-p_{B}$; this represents the baseline assumption that $E\left(p_{B}\right)=p_{A}$ and $E\left(p_{A}\right)=p_{B}$.

The calculations in (3) can be simplified by providing values of ${\mathrm{dz}}_{A,B}$ in a look‐up table indexed by references A and B, along with the sex and mean age of the target group.

Overweight needs to be defined *including obesity* for the algorithm to work properly—this is because the *z*‐score cut‐off defines the tail area of the distribution. So if overweight is net of obesity, then obesity prevalence needs adding to overweight prevalence.

### 2.3 Data

To test the algorithm two datasets were used. The Childhood Obesity Surveillance Initiative (COSI) study by Wijnhoven et al.^24^ gave paired prevalence rates of overweight and obesity based on the IOTF and WHO references, in 225 190 primary school boys and girls aged 6–9 years across 13 European countries during school year 2009/2010. The study provides paired prevalence rates for overweight (including obesity) and obesity in 52 distinct country–age–sex groups, with median 1358 individuals per group (mean 4331, IQR 1799, range 466–26 542), and grouped by age to the last completed year.

The study of Deren et al.^16^ gave prevalence rates of thinness, overweight (net of obesity) and obesity based on IOTF (thinness grade 1), WHO and CDC in 18 144 Ukrainian children and adolescents aged 6.5–17.5 years, grouped by sex and age to the nearest year. They allow the different prevalence rates to be compared in 22 distinct 6‐year groups. There were median 930 (range 145–1196) individuals per group, with up to 100 in the numerator per category per year at younger ages, but only one or two in the obese category at ages 15–17. For the analysis, obesity prevalence was added to overweight prevalence.

### 2.4 *Z*‐score differences

Table S1 lists values of ${\mathrm{dz}}_{A,B}$ by sex and age in half‐years from 2 to 18 years for all pairs of the thinness cut‐offs IOTF 16, IOTF 17, IOTF 18.5, WHO −2, WHO −1 and CDC 5, plus all pairs of the overweight and obesity cut‐offs IOTF 25, IOTF 30, IOTF 35, WHO +1, WHO +2, WHO +3, CDC 85 and CDC 95. For look‐up purposes, the age to use is that closest to the mean age of the target group.

### 2.5 An example

**FIGURE 1:**
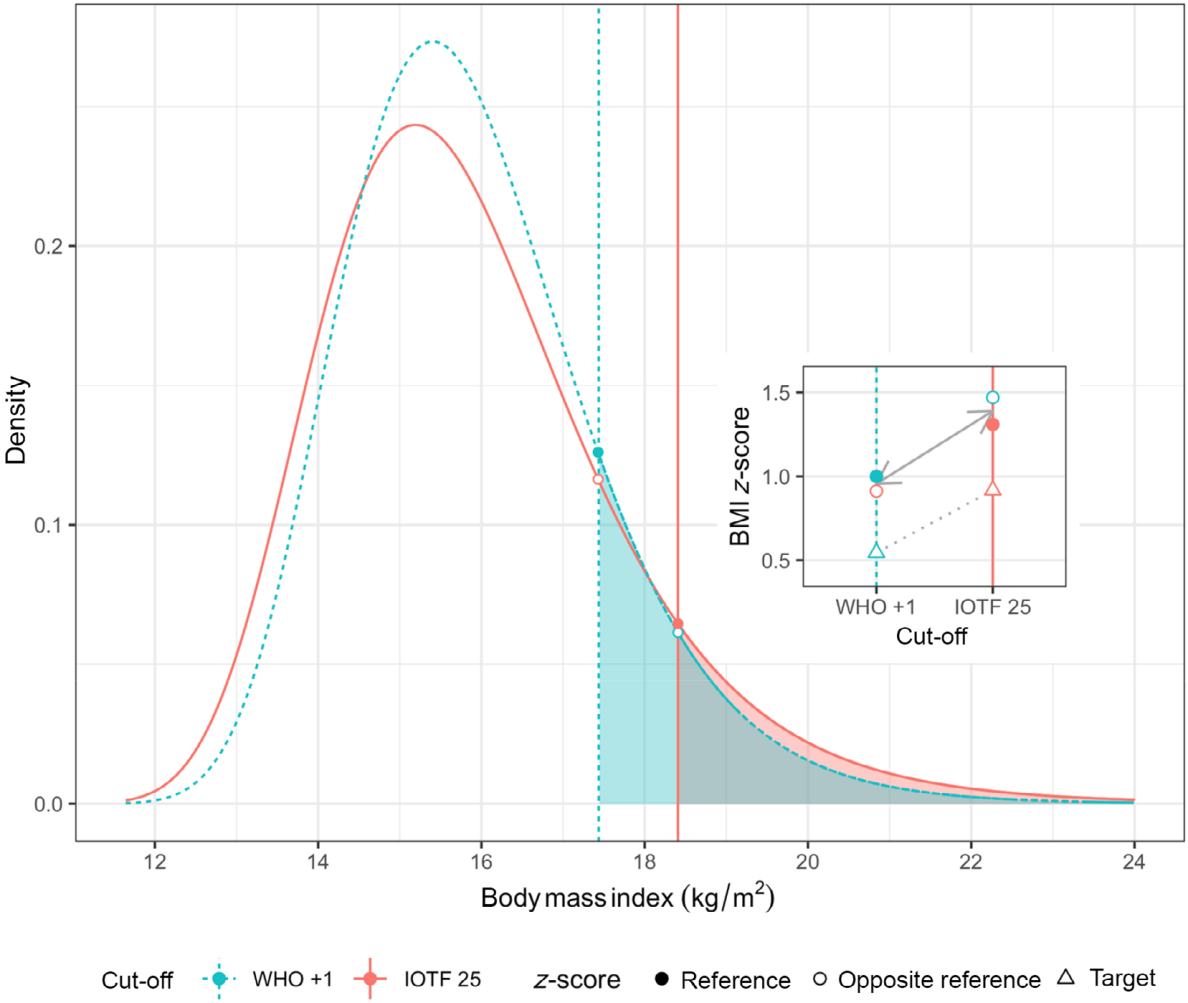
The frequency distribution of BMI in boys aged 8 according to the WHO and IOTF references. The vertical lines mark the overweight cut‐offs WHO +1 and IOTF 25, while the shaded areas indicate the corresponding overweight prevalence (grey where they overlap). The four points mark where the cut‐offs cross the distributions, with filled circles for the reference and open circles for the opposite reference. The inset shows the four points as BMI *z*‐scores according to the two references, along with open triangles for *z*‐scores corresponding to observed overweight prevalence in the target group of boys aged 8^16^

To illustrate the algorithm, Figure 1 uses as an example overweight prevalence (including obesity) in boys aged 8 according to WHO and IOTF. The figure shows (a) the skew reference BMI distributions for WHO (blue) and IOTF (red) as defined by their LMS parameters, (b) the WHO +1 and IOTF 25 BMI cut‐offs (vertical lines), and (c) the corresponding overweight prevalences defined as the area under the distribution curves beyond each cut‐off (shaded, with the area common to both in grey). This shows that because the IOTF cut‐off is higher than the WHO cut‐off, overweight is less common with IOTF.^2^, ^8^, ^16^

The points in Figure 1 mark where each vertical cut‐off crosses the two reference curves: its own reference (filled circles) and the opposite reference (open circles). Each point has a corresponding *z*‐score; for the filled circles ($z_{A}$ and $z_{B}$) the *z*‐scores are as defined in Table 1, that is, +1.00 for WHO and +1.31 for IOTF. For the open circles ($z_{A,B}$ and $z_{B,A}$) each *z*‐score is calculated for age and sex from the BMI cut‐off using the opposite reference; the WHO cut‐off of 17.4 kg/m^2^ for boys aged 8 corresponds to IOTF *z*‐score 0.91, while the IOTF cut‐off of 18.4 kg/m^2^ equates to WHO *z*‐score 1.47.

The inset to Figure 1 shows these four *z*‐scores plotted against the relevant BMI cut‐off, with a solid line joining the mean *z*‐scores on each cut‐off ($\bar{z_{A}}$ and $\bar{z_{B}}$), where ${\mathrm{dz}}_{A,B}=\bar{z_{B}}-\bar{z_{A}}=1.39-0.96=0.43$. In addition, the inset shows (as triangles) the overweight prevalence rates $p_{A}=29.3\%$ and $p_{B}=18.0\%$ for a target group of Ukrainian boys aged 8,^16^ plotted as *z*‐scores $Z_{A}=0.54$ and $Z_{B}=0.92$, respectively, and joined by a dotted line. The difference between them, $dZ_{A,B}=Z_{B}-Z_{A}=0.37$, is similar to ${\mathrm{dz}}_{A,B}=0.43$, as shown by the similar slopes of the dotted and solid lines. Applying (3) to $p_{A}$ and $p_{B}$ predicts prevalences of 31.5% for WHO and 16.4% for IOTF, as against the observed values of 29.3% and 18.0%, that is, errors of +2.2% and −1.6%—far less than the difference of 11.3% between the two prevalences.

### 2.6 Regression analysis

The algorithm (2) was fitted to the data as the probit regression equation:

(4) $$g\left(p_{B}\right)=Z_{A}+b\times {\mathrm{dz}}_{A,B}+\varepsilon$$

with link function $g\left(.\right)=-\mathrm{sign}\left(z_{B}\right)\Phi^{-1}\left(.\right)$. The coefficient b for ${\mathrm{dz}}_{A,B}$ defaults to 1, but tests for generalizability by tailoring the model to fit a particular dataset. The model omits the intercept to ensure reversibility, that is, exchangeability between A and B. The error term ε has two components: binomial error, that is, larger for small numbers, and error due to the size of ${\mathrm{dz}}_{A,B}$—the larger ${\mathrm{dz}}_{A,B}$ is, the greater the extrapolation and the larger the error. The model was fitted as an overdispersed beta binomial using GAMLSS^25^ with ε proportional to $\mathrm{abs}\left({\mathrm{dz}}_{A,B}\right)$. The interaction of ${\mathrm{dz}}_{A,B}$ with the data source and obesity‐overweight‐thinness was also fitted.

The models were fitted to the Wijnhoven^24^ and Deren^16^ data with overweight including obesity. Each of the 52 Wijnhoven sex–age groups provided prevalences for two A–B cut‐off pairs, that is, IOTF‐WHO for overweight and obesity. Each of the 22 Deren sex‐age groups provided prevalences for nine A–B cut‐off pairs: three each for thinness, overweight and obesity (i.e., IOTF‐WHO, WHO‐CDC and CDC‐IOTF). The models were fitted to all combinations of A–B cut‐off pairs and age–sex groups simultaneously, by ‘stacking’ the data into a single data frame with 52×2+22×9=302 rows. Each point is an A–B pair which can be viewed either as A predicting B or B predicting A; to avoid double counting the main analysis focused on pairs with positive ${\mathrm{dz}}_{A,B}$. Model fit was assessed using the Bayesian Information Criterion (BIC) and the residual SD (RSD) on the *z*‐score scale. The percentage of the variance of prevalence explained by the algorithm compared to the baseline model, where prevalence $p_{A}$ is predicted by $p_{B}$ and vice versa, was calculated as $100\left(1-{\left[\mathrm{SD}\left(p_{A}-E\left(p_{A}\right)\right)/\mathrm{SD}\left(p_{A}-p_{B}\right)\right]}^{2}\right)$ based on all 604 points.

The calculations were done in *R* (version 4.1.2) and GAMLSS (version 5.3‐4) running in RStudio (version 1.4). A function *ob_convertr* was written to do the conversion, which is available in the author's CRAN *sitar* package (version 1.2.0).^26^

## 3 RESULTS

### 3.1 *Z*‐score differences

Figure S1 shows ${\mathrm{dz}}_{A,B}$ the *z*‐score difference between cut‐off pairs WHO‐IOTF and CDC‐IOTF, and how it varies by sex and age, for overweight (dashed lines) and obesity (solid lines). The *z*‐score differences are larger for obesity than overweight; they are positive for most of childhood, and they fall with age. This confirms that the IOTF cut‐offs are generally higher than for WHO and CDC, particularly in early life.

Figure S2 shows the corresponding results for thinness, comparing WHO −2 and CDC 5 with IOTF grades 16, 17 and 18.5. Here ${\mathrm{dz}}_{A,B}$ has a wider range, it is more constant across age, and of the three IOTF grades, grade 2 (BMI 17 at age 18) is closest to the WHO and CDC cut‐offs.

### 3.2 Data visualization

**FIGURE 2:**
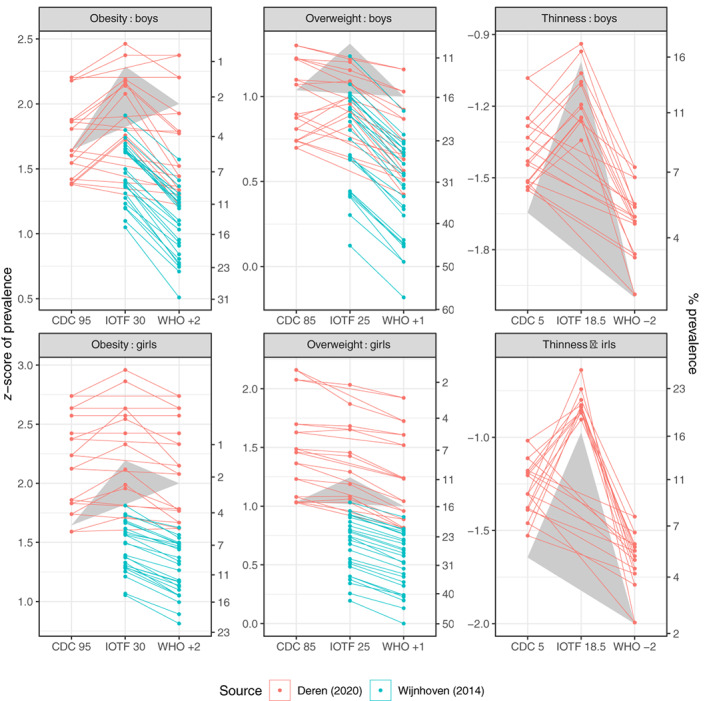
Prevalence rates by Centers for Disease Control, International Obesity Task Force and World Health Organization of obesity, overweight and thinness in groups of boys and girls from Deren^16^ (*n* = 22) and Wijnhoven^24^ (*n* = 52), on the *z*‐score scale (left) and the corresponding prevalence (%) scale (right). For overweight and obesity, the *z*‐score and prevalence scales are inversely related. The points for each group are connected by lines. The grey triangles correspond to the nominal prevalence rates defined by the three reference cut‐offs

Figure 2 shows, for the three references, prevalence rates of obesity, overweight and thinness by sex for the two datasets, on the *z*‐score scale (left axis) and the corresponding prevalence scale (right axis, note the nonlinearity). For overweight and obesity, the *z*‐score and prevalence scales are inversely related. The points for each group are connected by lines, where the slope of each line corresponds to the difference in prevalence $dZ_{A,B}$ on the *z*‐score scale between the two references, ranging from ~0 for the horizontal lines to >1 for IOTF 18.5 versus WHO −2 in girls. The background grey triangles correspond to the nominal prevalence rates defined by the three reference cut‐offs, for example, 16% for WHO +1 and 15% for CDC 85. The Figure shows a higher prevalence of overweight and obesity in Wijnhoven^24^ than Deren,^16^ and a lower prevalence for IOTF than WHO or CDC.

### 3.3 Fit of the algorithm

**FIGURE 3:**
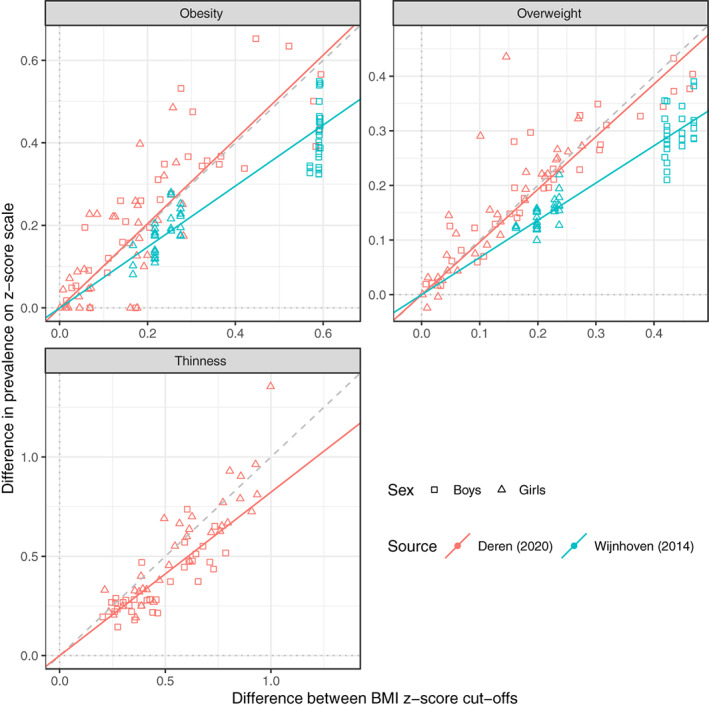
Differences in the prevalence of obesity, overweight and thinness, as measured on the *z*‐score scale, according to pairs of reference cut‐offs, plotted against the corresponding *z*‐score difference between the cut‐offs, in 74 groups of boys and girls aged 6.0–17.5^16^, ^24^ (*n* = 302). Each point corresponds to a line in Figure 2. The line of equality is shown (dashed), and points are coded by sex and data source, while regression lines per facet are coded by data source. The lines for obesity and overweight with Deren^16^ are close to the line of equality, while those for thinness and for Wijnhoven^24^ are not

Figure 3 tests the algorithm (2) by plotting $dZ_{A,B}$ the *z*‐score difference in prevalence versus ${\mathrm{dz}}_{A,B}$ the *z*‐score difference between cut‐offs. Obesity, overweight and thinness are shown separately, coded by sex and source. Each point corresponds to one of the 302 lines in Figure 2, with the slope ${\mathrm{dz}}_{A,B}$ selected to be positive. Overall $dZ_{A,B}$ is highly correlated with ${\mathrm{dz}}_{A,B}$ across the three facets (r=0.88). But the more important question is whether $dZ_{A,B}$ and ${\mathrm{dz}}_{A,B}$ are on average the same, that is, that the points are scattered symmetrically along the line of equality. This line is shown dashed, and the overweight and obesity data for Deren^16^ (in red) lie along it. However, the data for Wijnhoven^24^ (in blue) do not; they lie along a line appreciably shallower than the line of equality. The fitted regression lines through the origin for the two datasets have highly significantly different slopes.

Table 2 summarizes three models fitted to the data. Model #1 corresponds to the algorithm and assumes that the data lie on the line of equality, that is, that b=1 in (4), while model #2 estimates b=0.78 in (4), which materially reduces the BIC and RSD. Model #3 corresponds to the five regression lines in Figure 2, with the BIC and RSD further reduced, where the slope confidence intervals for Deren^16^ and Wijnhoven^24^ are non‐overlapping. Because the Wijnhoven sample is much larger, the combined slope in model #2 is closer to the Wijnhoven slope than the Deren slope in model #3. To weight the two data sources equally, the five coefficients in model 3 are averaged to give b=0.84.

**TABLE 2:** Summary of beta binomial regression models of prevalence fitted to the Deren and Wijnhoven data (*n* = 302). Estimates of the regression coefficient b for ${\mathrm{dz}}_{A,B}$ in (4)

Model	Term in (4)	Regression coefficient b (95% CI)	BIC	Residual SD
1	Line of equality	1 (fixed)	2487	0.110
2	Overall	0.78 (0.75–0.80)	2303	0.095
3	Deren^16^ obesity	1.02 (0.92–1.12)	2223	0.083
	Deren^16^ overweight	0.96 (0.90–1.02)		
	Deren^16^ thinness	0.82 (0.78–0.86)		
	Wijnhoven^24^ obesity	0.74 (0.70–0.78)		
	Wijnhoven^24^ overweight	0.68 (0.65–0.70)		

Abbreviations: BIC, Bayesian Information Criterion; CI, confidence interval.

**FIGURE 4:**
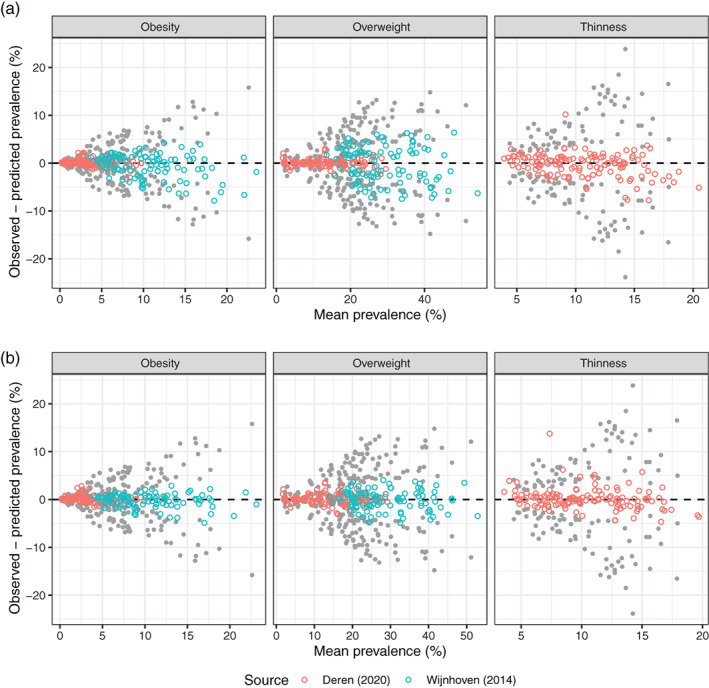
Bland–Altman plots comparing observed and predicted prevalence (%) of obesity, overweight and thinness, colour‐coded by data source (*n* = 604), with predicted prevalence calculated in two ways: (A) from observed prevalence and ${\mathrm{dz}}_{A,B}$ (3); and (B) as for (A) except that ${\mathrm{dz}}_{A,B}$ is multiplied by $b_{1}=0.84$. Also shown (in grey) are Bland–Altman plots comparing the original prevalence data for the pairs of references. The scatter about the origin of the original data is greatly reduced by applying the algorithm, and more so for (B) than for (A)

Figure 4 shows Bland–Altman plots comparing observed and predicted prevalence (%) of obesity, overweight and thinness. For each pair of prevalences, the difference between them, that is, the residual, is plotted against their mean. The data are colour‐coded by data source including both positive and negative ${\mathrm{dz}}_{A,B}$ (*n* = 604), with predicted prevalence calculated in two ways: Figure 4A from observed prevalence and ${\mathrm{dz}}_{A,B}$ (3); and Figure 4B as 4a except that ${\mathrm{dz}}_{A,B}$ is multiplied by b=0.84, the mean slope in model #3. Ideally the points should lie along the horizontal dashed line, which broadly they do, with Deren (in red) fitting better than Wijnhoven (in blue). In addition, the Wijnhoven points fit better in Figure 4B than 4a, while for Deren they are slightly worse.

Figure 4 also shows the underlying prevalence rates $p_{A}$ and $p_{B}$ as grey points, plotted as their difference $\pm \left(p_{A}-p_{B}\right)$ versus their mean $\left(p_{A}+p_{B}\right)/2$. This corresponds to the baseline model where prevalence $p_{A}$ predicts $p_{B}$ and vice versa. The points are symmetric about the x‐axis, and they fan out from the origin. The figure shows how the algorithm ‘shrinks’ the residuals by shifting the grey points towards the coloured points. Table 3 compares the SDs of the residuals under the baseline and fitted models. Overall the algorithm explains more than 88% of the baseline variance (Figure 4A), and more than 94% when adjusted for bias (Figure 4B).

**TABLE 3:** The residual SD (%) of prevalence for obesity, overweight and thinness, that is the SD of observed minus predicted prevalence, under three models: (i) baseline, where $p_{A}$ predicts $p_{B}$ and vice versa; (ii) algorithm (3); and (iii) bias‐adjusted algorithm (4)

Model	Obesity (*n* = 236)	Overweight (*n* = 236)	Thinness (*n* = 132)	All (*n* = 604)
(i) Baseline RSD (%)	4.5	6.6	9.2	6.6
(ii) Algorithm RSD (%)	1.8	2.6	2.5	2.3
Variance explained (%)	84.3	85.0	92.8	88.2
(iii) Adjusted algorithm RSD (%)	1.1	1.6	2.2	1.6
Variance explained (%)	94.3	94.3	94.1	94.2

*Notes*: The percentage of the baseline variance explained by the algorithm is also shown. The algorithm explains 88% of the baseline variance, increasing to 94% when adjusted for bias.

## 4 DISCUSSION

The study has explored the feasibility of a reversible and generalizable algorithm to convert prevalence rates for thinness, overweight and obesity from one BMI reference to another. The algorithm assumes that the difference in prevalence attributable to the two references can be explained as a shift on the underlying BMI *z*‐score scale, where the magnitude of the shift depends on the two references, and the age and sex of the group of children being assessed.

The algorithm is made easier to implement by providing the relevant *z*‐score shift in a table indexed by the two references, age and sex. However, the algorithm's performance depends critically on the assumption that the difference in prevalence on the *z*‐score scale truly is equal to the *z*‐score difference between the cut‐offs.

Figure 3 tests this assumption by plotting prevalence difference against cut‐off difference, and if true the data ought to be distributed symmetrically along the line of equality, indicating a lack of bias. The data of Deren^16^ (in red) follow this pattern, the regression lines for obesity and overweight being close to the line of equality, with regression slope confidence intervals including one (Table 2 model #3). However for Wijnhoven^16^ the data are biased, departing materially from the line of equality, with regression slopes of around 0.7 with tight confidence intervals. This demonstrates that the algorithm can be unbiased or biased, depending on the dataset; with Deren it is reversible while with Wijnhoven it is not.

So given this, how well does the algorithm perform? It provides a coherent way to convert prevalences from one reference to another, but the key issue is whether the associated prediction error is small enough for the algorithm to be useful. Figure 4A shows that the algorithm explains 88% of the variance in prevalence between the pairs of references, which is substantial. Thus one can use the algorithm as it stands, that is, viewing it as generalisable, and obtain useful estimates of prevalence. However, the prediction error of 12% is inflated due to heterogeneity between datasets.

Alternatively one can use the bias‐adjusted algorithm, with ${\mathrm{dz}}_{A,B}\times 0.84$ rather than ${\mathrm{dz}}_{A,B}$ in (3). This explains a substantial 94% of the variance and halves the prediction error to 6% (Figure 4B). However, the coefficient of 0.84 is based on just the two datasets and cannot be assumed universally representative—it requires validation.

So in summary there is a trade‐off between wider generality (using the algorithm unadjusted) and better fit (adjusting the algorithm for bias), and the key question is, ‘Which is more useful?’. There is a paper in preparation which describes a materially improved version of the algorithm.

Why should the regression slope in Figure 3 be less than one? It means that shifting from one cut‐off to a higher cut‐off (positive ${\mathrm{dz}}_{A,B}$, e.g., from WHO to IOTF) the algorithm underestimates the true prevalence of overweight and obesity (and exaggerates it for negative ${\mathrm{dz}}_{A,B}$), which means that the target BMI distribution has a heavier upper tail than the reference distribution, as measured on the *z*‐score scale—it indicates a secular trend to increasing skewness, as has been documented for US data.^27^ The heavier tail may be due to the LMS method's S (coefficient of variation) being larger, and/or L (Box‐Cox power) being smaller.^22^

The reversibility of the algorithm is useful for systematic reviews of child obesity prevalence. The researcher can choose which reference—IOTF, WHO or CDC—to use as their baseline, and then apply the algorithm to convert prevalences based on the other references to the baseline prevalence. Researchers in the future may want to cite these prevalences, but first rebasing them to a different reference, and this can be done with no loss of information.

Deren et al.^16^ chose to use IOTF grade 1 (BMI 18.5 at age 18) to define thinness. However, Table 1 and Figure S1 show that IOTF grade 2 (BMI 17 at age 18) is closer to the WHO and CDC cut‐offs, and hence would need less extrapolation in the conversion. Grade 2 is also the grade recommended for use in the original IOTF paper.^7^ This indicates that thinness conversion should work better with IOTF grade 2 than grade 1, the cut‐offs being closer together.

The study has some limitations. The two example datasets provide only a glimpse of how the algorithm might work in practice, and other studies are needed to apply it to other data. Also, Deren^16^ and Wijnhoven^24^ used different definitions of the IOTF cut‐offs, respectively, reference 6 and reference 8, though the two are very similar in practice.

The study also has strengths. The algorithm is theoretically based and is reversible, although arguably not generalizable. It has been applied to BMI and the IOTF, WHO and CDC reference cut‐offs. But it is sufficiently general that it can be applied to *any* anthropometric reference, with *any* measurement, where the cut‐offs are defined as *z*‐scores and can be back‐transformed to measurement units. This provides an opportunity to exploit for example the historical literature of overweight and obesity prevalence data based on locally developed BMI references.^28^ The algorithm is likely to fit better with older datasets, as the duration of any secular trend to greater obesity will have been shorter.

In conclusion, the paper describes an algorithm for converting between prevalence rates of overweight, obesity and thinness based on the IOTF, WHO and CDC BMI reference cut‐offs. Applied to two example datasets the algorithm performs well, and its reversibility makes it a useful tool for harmonizing prevalence rates across references.

A follow‐up paper in preparation describes an improved version of the algorithm, which is both reversible and generalisable.

## CONFLICT OF INTEREST

Tim J. Cole declares the following conflicts of interest: he developed the LMS method with Peter Green^22^ and was first author on papers describing the IOTF cut‐offs.^6^, ^7^, ^8^ Tim Lobstein was also an author on the latter paper.^8^

## AUTHOR CONTRIBUTIONS

Tim J. Cole designed the study, did the data analysis, generated the figures and wrote the first draft of the paper. Tim J. Cole and Tim Lobstein edited the paper and had final approval of the submitted and published versions.

## Supporting information


**Table S1.** See separate Microsoft Excel spreadsheet.

**Figure S1.** The distance between cut‐offs for IOTF compared to WHO and CDC, expressed as *z*‐score differences by sex and age, for overweight and obesity.
**Figure S2**. The distance between thinness cut‐offs for IOTF grades 1, 2 and 3 compared to WHO −2 and CDC 5, expressed as *z*‐score differences by sex and age.
