# Supplementary material for: Exploring an algorithm to harmonize International Obesity Task Force and World Health Organization child overweight and obesity prevalence rates
Source: Pediatr Obes. 2022 Feb 22;17(7):e12905. doi: 10.1111/ijpo.12905 (PMC9285550; doi:10.1111/ijpo.12905)
Supplement: Supplementary file 2 — Figure S1. The distance between cut‐offs for IOTF compared to WHO and CDC, expressed as z‐score differences by sex and age, for overweight and obesity. Figure S2. The distance between thinness cut‐offs for IOTF grades 1, 2 and 3 compared to WHO −2 and CDC 5, expressed as z‐score differences by sex and age. [file IJPO-17-0-s001.docx]

Supplementary Figure 1. The distance between cut-offs for IOTF compared to WHO and CDC, expressed as z-score differences by sex and age, for overweight and obesity.

Supplementary Figure 2. The distance between thinness cut-offs for IOTF grades 1, 2 and 3 compared to WHO -2 and CDC 5, expressed as z-score differences by sex and age.
